# Comparing Pregnant and Postpartum Client and Provider Feedback on a Digital Health Intervention for Substance Use Recovery: User-Centered Design Approach

**DOI:** 10.2196/86255

**Published:** 2026-03-09

**Authors:** Hannah Szlyk, Layna Paraboschi, Lucy Meigs, Elecia Worley, JaNiene Peoples, Emily Maranets, Erin Kasson, Alex Ramsey, Corey Lau, Kristin Korte, Patricia Cavazos-Rehg

**Affiliations:** 1 Department of Psychiatry Washington University School of Medicine St Louis, MO United States; 2 College of Social Work Florida State University Tallahassee, FL United States; 3 Healthy Brain and Child Development Study Washington University School of Medicine St Louis, MO United States; 4 Clinic for Acceptance, Recovery, and Empowerment in Pregnancy Barnes Jewish Hospital St Louis, MO United States

**Keywords:** pregnant people, substance use disorders, user-centered design, mHealth, mobile health, postpartum

## Abstract

**Background:**

Mobile health (mHealth) interventions can expand access to and engagement in lifesaving treatment for pregnant and postpartum people with a substance use disorder. Yet, many people with lived experience and substance use providers alike are often excluded from mHealth intervention development, limiting opportunities to provide feedback on critical design components such as usability, cultural relevance, and compatibility with real-world practice.

**Objective:**

The study engaged pregnant and postpartum people and substance use providers in a formative evaluation to refine an mHealth intervention designed to support recovery.

**Methods:**

Pregnant and postpartum participants (n=11) and providers working in recovery settings (n=13) across Missouri reviewed the same mHealth intervention. Participants completed a survey and semistructured qualitative questions on usability and compatibility after reviewing the same mHealth intervention. Survey responses and qualitative themes were compared across groups. Post hoc analyses examined differences between pregnant and postpartum participants who had used the app and those who had not (n=8) to identify barriers to participation.

**Results:**

Both participant groups reported similar themes related to the usability and compatibility of the mHealth intervention, including a need for simplified navigation and greater personalization of app content. The e-coaching feature and directory of recovery-focused resources were viewed as valuable by both groups. Uniquely, pregnant and postpartum participants emphasized the need for app content addressing craving management, emotional triggers, and parenting stress. These participants also requested more frequent communication with the e-coach than providers recommended. Nonapp users differed from app users by race, education, and household characteristics, underscoring structural barriers to engagement.

**Conclusions:**

Engaging both pregnant and postpartum people and providers in formative evaluation reveals overlapping and distinct priorities for mHealth design. Findings highlight that user-informed development is essential for improving usability, engagement, and recovery outcomes, including reaching those least likely to engage with traditional or digital treatment supports.

## Introduction

### Background

Substance use and related overdoses remain leading causes of perinatal death in the United States [1], particularly during the postpartum period [2,3]. Most deaths among people with substance use disorder (SUD) involve the use of opioids and stimulants [4]. Initiation of substance-use recovery-focused perinatal care is the optimal strategy to reduce fatal outcomes related to substance use [5]. Through treatment, individuals can learn about the impact of substance use on themselves and their baby and can receive prescriptions for medications such as buprenorphine and methadone to reduce cravings and symptoms [6]. Despite the availability of current evidence-based interventions, pregnant and postpartum people with SUD face multiple barriers to accessing and staying on SUD treatment. These include unavailability of treatment services, limited access to specialized care, lack of social support to support recovery [7], and comorbid mental health conditions related to substance use. These can decrease rates of treatment retention and usually exacerbate SUD symptoms [8]. Pregnant and postpartum people may also prioritize the health of their baby and have less time to consider treatment for their own health and recovery [9]. Furthermore, stigma, criminalization, and the threat of child-protective services can deter individuals from seeking care in the first place [10].

Mobile health (mHealth) can potentially help pregnant and postpartum people access and stay engaged in treatment. mHealth is the use of social media, phones, and other technology (eg, app-based interventions) to measure and improve health outcomes [11]. mHealth tools can bridge gaps in treatment linkage and use by providing reminders for upcoming appointments, sending notifications for medications, and helping to provide “between appointment” support. The use of mHealth tools has also been shown to have great potential for supporting health care providers [12]. For example, mHealth interventions may help to alleviate the burden of a high caseload for health care providers by sharing or taking on tasks such as education and resource dissemination or providing emotional and social support. There is also the potential for health care providers to receive updates about their clients’ well-being and progress through remote strategies, versus only at in-person appointments, using mHealth tools [13].

Health care providers who are at the frontline of clinical care can play an important role in mHealth development and refinement and are considered integral to the user-centered mHealth design process. User-centered design is a process that refers to how key informants and end users including clients and health care providers influence the design of a tool through involvement in the design process [14]. The involvement of both health care providers and SUD clients in the user-centered design process is key to ensure that a final product actually meets the needs of clients and can be easily integrated into pathways of care by providers. Reliance on the feedback of one key informant group could hinder the success of the final product (eg, increasing access to SUD and prenatal care), especially as providers and clients may not always be aligned in their preferences and priorities for care. Yet, there is a critical gap in mHealth interventions for pregnant and postpartum people with SUD that includes input from people with lived experience [15,16], providers, or both [17] in the design process.

Usability and compatibility are key areas of user experience and technology adaptation. Usability explores how an mHealth tool is technically effective (desired outcomes or goals are achieved), efficient (can be used easily and quickly), and satisfactory (is likable and meets expectations) [18,19]. Compatibility assesses how an mHealth tool fits the targeted end user, such as if the tool is appropriate for the user’s stage in recovery and daily life as a pregnant person or new parent with SUD [20]. Applying user-centered design and mHealth testing can reveal unasked but necessary questions about how to support this specific population and provide insight into current changes in the local recovery landscape. Leveraging user-centered design approaches could greatly enhance the translation of evidence-based practices from research to tailored mHealth supports for pregnant and postpartum people with SUD [14].

### This Study

The study sought to engage pregnant and postpartum people and health care providers in formative evaluation, including usability testing, to guide further mHealth app refinement and to support recovery from SUD. Our primary questions were as follows: (1) What are the perceptions of pregnant and/or postpartum clients regarding an mHealth intervention developed to support their SUD recovery during the pre- and postnatal stages? (2) What are health care provider perceptions of an mHealth intervention to support clients with SUD during pregnancy and the postpartum period? (3) How are client and provider perceptions about an mHealth intervention and barriers to care similar or different? To triangulate key informant perspectives, we conducted post hoc analyses of the barriers to participation reported by pregnant and postpartum clients who did not use the app. Findings contribute to the needed literature on feedback from multiple key informants, particularly people with lived experience, in the design and refinement of digital health interventions for SUD.

## Methods

### Overview

This study examines the usability and acceptability of an mHealth intervention (uMAT-R) among pregnant and postpartum people after 1 week of app use and is part of a larger, ongoing intervention. uMAT-R is pronounced as “you matter” and is based on the acronym for medication-assisted treatment. Please see Eswaran et al [21] and Szlyk et al [22] for more information about the parent intervention project, including intervention effectiveness and participant recovery outcomes. uMAT-R is a mobile app with features originally designed to educate people who use opioids and/or stimulants and includes tailored content to support recovery during pregnancy and the postpartum period (Multimedia Appendix 1). Over the past few years, the research team has conducted an iterative user-centered design program of research to increase uptake and continued engagement with the uMAT-R app. The app contains educational content (a course library) that is designed to counter misconceptions and deficits in medication for opioid use disorder knowledge and to support a healthy pregnancy, postpartum recovery, and overall recovery capital (eg, employment and housing) [23]. In the app, participants have access to a recovery e-coach that follows a motivational interviewing model [24]. Participants have access to the e-coach during weekdays and general workday hours (eg, 8 AM-6 PM) for approximately 1 month. e-Coaches serve as an additional support to clients and are trained in person-centered therapy techniques and crisis intervention. e-Coaches help clients set goals and provide information on community resources. Pregnant and postpartum participants can also set their own goals, create reminders, and use a sober day tracker within the uMAT-R app. Finally, the uMAT-R app contains a resource directory with information on where to find support with housing, food, clothing, employment, and specific services for pregnant and postpartum women and people and their families. The following sections describe the specific enrollment and consenting processes conducted with each participant group as part of this formative work.

### Pregnant and Postpartum Clients

Eligible client participants (these individuals will be referred to as “clients” throughout this paper) included those who were aged 18 years or older, had a history of opioid or stimulant use, were receiving treatment at a consenting treatment facility and/or referred from other participants or a community partner, were pregnant (second or third trimester) or postpartum within the past 3 years, were US residents, and owned a smartphone with an iOS or Android operating system. Eligible clients were invited to provide initial feedback after using the uMAT-R app for 1 week. Data collection occurred from July 2022 through December 2024. Clients were asked to complete a (1) baseline survey and (2) open-ended app usability questions at week 1. Initially, clients were asked to complete the week 1 questions via a phone or Zoom (Zoom Video Communications) interview. After having trouble with scheduling and completing the interviews using this approach, the research team decided to send clients the open-ended questions in a survey format via REDCap (Research Electronic Data Capture; Vanderbilt University). This strategy appeared to better suit the needs of the clients, as they balanced recovery and family life.

In total, 16 clients logged on to the app and initiated the open-ended questions; 11 of these clients completed the 31 open-ended questions at week 1. Clients who logged into the app took between 10 and 30 minutes to complete all the questions as indicated by the REDCap time stamp. An additional 8 clients did not log on to the app and completed 11 open-ended questions about app use in general and identified barriers to using this app. Time spent completing the questions ranged from 3 to 10 minutes. Our main analysis will focus on the 11 clients who logged on and answered open-ended questions. Our post hoc analysis includes the 8 clients who answered the open-ended questions about barriers to app use but did not log into the app.

### Health Care Providers

Providers from regional treatment facilities in both Missouri and neighboring Illinois were recruited via email or telephone for enrollment. Additional providers were recruited via study announcements circulated through regional and state-specific listservs for health care providers and researchers who support individuals in recovery from SUD, including during pregnancy and/or the postpartum period. One listserv included a regional network of health care providers and staff who receive funding through the same Substance Abuse and Mental Health Services Administration grant. Some providers were recruited via snowball sampling (eg, referrals to our study). Individuals who expressed interest in participating were sent up to 3 email reminders about the study and next steps for enrollment.

In total, 22 health care providers responded to the study flyers and expressed interest in the study. Of those individuals, 16 providers agreed to participate in the study and provided consent. Overall, 16 providers completed the brief survey; 13 completed the follow-up interview.

### Ethical Considerations

The Washington University Institutional Review Board (IRB) approved the study (IRB study ID: 2021022070). Quantitative surveys, including open-ended written questions, were recorded using the REDCap survey tool, which is a secure, web-based application designed to support data capture for research studies that are approved by the IRB. Interview audio and transcripts were stored on the secure platform, Box, which is also approved for storage of human subjects’ data by the IRB. All study participants were granted a study ID, which was used to label participants’ survey responses and interviews. The study log including the participants’ identifiable information was stored separately from the raw data, also on Box. Only study personnel had access to the study data and study log. These procedures assisted the study team in upholding participant privacy and confidentiality. Clients provided written, informed consent when they were enrolled in person at recruitment activities that took place at participating treatment facilities. If a client contacted the study team at the laboratory phone number, the participant received the study information over the phone and could provide consent verbally. Clients were compensated US $20 for completing the baseline survey and US $10 for completing the open-ended survey questions. Participating providers gave verbal consent to participate in the study, including to be interviewed and recorded (audio and video). Enrollment was designed to be brief and less intrusive for the standard 9-5 workday (eg, a 15-minute phone call at participants’ desired time). Participants were compensated with a US $20 e-gift card after completing a brief survey and a 30- to 45-minute feedback interview.

### Qualitative Data Collection

Open-ended questions, either completed via survey or interview, were conducted among clients and health care providers approximately 1 week after participants engaged with the mHealth intervention. Providers could interview in person, via telephone, or via Zoom, and interviews lasted approximately 30-45 minutes. Following a user-centered design approach, the questions assessed characteristics of usability (efficiency, technical effectiveness, and satisfaction) and compatibility (eg, the app’s suitability of the daily schedule as a pregnant person or new parent and the recovery status of the user) of uMAT-R, informed by the International Organization for Standardization [25]. The questions assessed participants’ engagement with the app (or barriers to engagement) and their recommendations for mHealth use to treat SUD. Open-ended survey questions were collected in REDCap. Each interview was audio-recorded and transcribed. A team member completed interview summaries after each interview to provide an audit trail of the qualitative research process and to ensure fidelity to the interview guide [26]. The interview summaries documented characteristics of the interview, including rapport and whether any interviewer biases emerged. Interview summaries were not returned to participants.

### Data Analysis

#### Qualitative Data Coding and Analysis

The primary coders (including coauthor EM) were members of a clinical research team, who support the larger parent study. Using an iterative process of content analysis [27], the 2 pairs of team members coded transcribed interviews and survey responses using a codebook based on question domains from the original interview guide. Since both participant groups were asked the same questions, a single codebook was developed for the study. Example codes included “education videos,” “preferred format,” and “use of goals features” (for clients). Microsoft Excel software was used for coding. Meaningful units or quotes [28] from each case were pasted under each code’s column. Next, the team consolidated initial codes into broader categories that more accurately explained the data (eg, “what to change within the app” and “motivators to use the app”), and the 2 team members recoded condensed units of text using this updated codebook. In total, 3 coders could not continue with the project (eg, left the laboratory to continue graduate education), and 3 additional team members took their place to support the analyses. The new coders (including coauthors JP, LM, and LP) were able to view how their past teammates had coded the text within the Excel spreadsheets, including disagreements about codes. The 2 new pairs (one pair assigned to provider transcripts; the second pair assigned to pregnant and postpartum participant transcripts) met periodically to discuss differences in coding. The principal investigator (HS) served as the third coder for both teams to help identify areas of coding inconsistency. The 2 pairs of coders and the principal investigator (HS) met to review inconsistencies and resolve discrepancies until 100% consensus was obtained [29].

Multimedia Appendix 2 shows how the full team evaluated study decisions by each of the 4 criteria for trustworthiness based on the work of Lincoln and Guba [30]: credibility, transferability, dependability, and confirmability. To further enhance the trustworthiness of the findings, the analytical team identified and examined 2 negative cases. Negative cases are defined as those that deviate from the main themes identified in a study [31], and their presence can help to better understand the potential limitations and variability of findings and their application. Only 1 client suggested that the mobile app should be more colorful, and this same client shared that they would not like to use the app to track recovery goals, as they preferred to write down this type of information in a journal. Another client reported that they would have been more motivated to use the app if they had been in an earlier phase in their recovery, when they would have needed more guidance and support. Potential implications in the context of the main findings are explored in the Discussion section.

#### Quantitative Analysis

Quantitative survey data and demographic data were summarized by group using descriptive statistics. Both clients and providers completed questions via a REDCap survey about personal sociodemographics and barriers to care at the beginning of the study. Items about barriers to care were slightly different between the 2 groups. Clients were asked to select reasons for missing any SUD treatment or recovery service appointments in the past month. This question was adapted from an earlier study that examined barriers to treatment among Mexican American and European American women with eating disorders [32]. The constructs and response options featured closely resemble that of the SUD treatment barrier question from the National Survey on Drug Use and Health [33]. Similarly, providers were asked to identify significant barriers to care that their clients face. This question was developed by the authors’ research laboratory as part of an alternative project to assess the experience of SUD treatment providers during COVID-19 [34]. Post hoc, survey data from clients who were inactive in the app were analyzed to determine any demographic differences between these clients and those who were able to use the app.

## Results

### Survey Results

#### Active Pregnant and Postpartum Clients

In total, 11 pregnant and postpartum client participants engaged with the uMAT-R app and completed the baseline survey (Table 1). At the time of the study, 6 participants were pregnant, and 5 were postpartum. A total of 10 clients identified as women; 1 client identified as gender nonconforming or nonbinary. Of these clients, 1 identified as Black or African American, 1 identified as Hispanic, 6 identified as White, and 3 belonged to other racial or ethnic groups. The highest level of education achieved for over half of the clients was a General Educational Development test or a high school diploma. Most of the clients (8/11) were unemployed and covered by Medicaid. In the past 12 months, 8 clients reported having more than 1 phone. Most clients (10/11) reported currently being in treatment for SUD, and 6 clients reported having missed appointments in the past month. In total, 5 clients were in recovery from opioid use, 5 were in recovery from dual opioid and stimulant use, and 1 client was in recovery from stimulant use only. Only 3 clients reported being prescribed medication for opioid use disorder (MOUD). When asked about reasons for missed appointments in the past month, the top response was not having transportation (3/11).

**Table 1:** Pregnant and postpartum active participant demographics (n=11).

		Values, n (%)
**Age range (years)**		
	18-30	8 (72.7)
	31-50	3 (27.3)
**Gender identity**		
	Women	10 (90.9)
	Nonbinary	1 (9.1)
**Race or ethnicity**		
	Black or African American	1 (9.1)
	Hispanic	1 (9.1)
	White	6 (54.5)
	Other	3 (37.3)
**Education level**		
	GED^a^ or high school diploma	6 (54.5)
	Some college or higher	5 (45.5)
**Employment status**		
	Employed full-time	2 (18.2)
	Employed part-time	1 (9.1)
	Unemployed	8 (72.7)
**Pregnant or postpartum**		
	Pregnant	6 (54.5)
	Postpartum	5 (45.5)
**Substance use**		
	Opioids	5 (45.5)
	Stimulants	1 (9.1)
	Opioids and stimulants	5 (45.5)
**MOUD^b^**		
	Prescribed MOUD?^c^	3 (27.3)
	Buprenorphine or suboxone	1 (9.1)
	Methadone	2 (18.2)
**Health insurance**		
	Medicaid	8 (72.7)
	Private health insurance	1 (9.1)
	No insurance	2 (18.2)
**Number of children**		
	1	5 (45.5)
	2	3 (27.3)
	3	2 (18.2)
	4+	1 (9.1)
**Number of phones in the past 12 months**		
	1	3 (27.3)
	2	4 (36.4)
	3+	4 (36.4)
**Number of phone numbers in the past 12 months**		
	1	3 (27.3)
	2	5 (45.5)
	3+	3 (27.3)
**Treatment status**		
	Currently in treatment	10 (90.9)
	Not currently in treatment	1 (9.1)
**Any missed appointments in the past month**		
	Yes	6 (54.5)
	No	5 (45.5)

^a^GED: General Educational Development test.

^b^MOUD: medication for opioid use disorder.

^c^Only 3 participants responded to this question.

#### Health Care Providers

In total, 13 providers completed both the survey and the interview (Table 2). Of these providers, 9 were identified as women. Only 1 was American Indian, 2 were Black or African American, 9 providers were White, and 1 identified as belonging to another racial or ethnic group. A total of 3 individuals worked as peer providers, 2 as community health workers, 3 as program administrators, 2 as medical doctors, and 3 as behavioral health technicians. Nearly half (6/13) of the providers reported being in recovery from SUD themselves.

**Table 2:** Health care provider demographics (n=13).

Characteristic			Values, n (%)
**Age range** (years)			
	18-30	2 (15.4)	
	31-50	9 (69.2)	
	51+	2 (15.4)	
**Gender**			
	Women	9 (69.2)	
	Men	3 (23.1)	
	Genderqueer	1 (7.7)	
**Race**			
	American Indian	1 (7.7)	
	Black or African American	2 (15.4)	
	White	9 (69.2)	
	Other	1 (7.7)	
**Ethnicity**			
	Non-Hispanic or Latino	12 (92.3)	
	Hispanic or Latino	1 (7.7)	
**Education**			
	GED^a^ or high school diploma	4 (30.8)	
	Some college credit	2 (15.4)	
	Bachelor degree	4 (30.8)	
	Master degree	1 (7.7)	
**Role at work**			
	Peer	3 (23.1)	
	Community health worker	2 (15.4)	
	Administrator	3 (23.1)	
	Medical doctor or psychiatrist	2 (15.4)	
	Behavioral health technician	3 (23.1)	
**Place of work**			
	Recovery community center	7 (53.8)	
	Hospital-based setting	3 (23.1)	
	Other	3 (23.1)	
**In recovery themselves**			
	Yes	6 (46.2)	
	No	7 (53.8)	

^a^GED: General Educational Development test.

When asked about barriers to treatment for pregnant and postpartum clients (Table 3), the top reasons reported by providers included lack of transportation (12/13), low motivation to stay in recovery (12/13), a lack of childcare (11/13), feelings of stigma from others (11/13), relapse (10/13), and fears of being reported to social services (9/13).

**Table 3:** Barriers to care for people in treatment for opioid use disorder during the postpartum period based on health care provider perspectives (n=13).

Barriers to care		Values, n (%)
**Lack of transportation**		
	Yes	12 (92.3)
	No	1 (7.7)
**Lack of appropriate technology**		
	Yes	6 (46.2)
	No	7 (53.8)
**Does not like sessions**		
	Yes	3 (23.1)
	No	10 (76.9)
**Has relapsed**		
	Yes	10 (76.9)
	No	3 (23.1)
**Low motivation to stay in recovery**		
	Yes	12 (92.3)
	No	1 (7.7)
**Lack of childcare**		
	Yes	11 (84.6)
	No	2 (15.4)
**Cost of appointment**		
	Yes	5 (38.5)
	No	8 (61.5)
**Length of appointment**		
	Yes	2 (15.4)
	No	11 (84.6)
**Lack of health insurance**		
	Yes	4 (30.8)
	No	9 (69.2)
**Fear of being reported to social services**		
	Yes	9 (69.2)
	No	4 (30.8)
**Feelings of stigma from others**		
	Yes	11 (84.6)
	No	2 (15.4)
**Having to work^a^**		
	Yes	8 (61.5)
	No	4 (30.8)

^a^One participant did not answer this question. Two participants responded to an additional question about which barriers were missed. They suggested a lack of scheduling skills, a lack of drug-free housing, and a shortage of treatment.

### Qualitative Results

#### Overview

The themes and minor themes for both key informant groups per category are described below, with representative quotes for both providers and clients shown in Tables 4 and 5.

**Table 4:** Themes: uMAT-R feedback from providers and clients.

Categories	Theme and quote	
	Provider	Client
What to change within the app	Streamline content and information: “But there is a lot, yeah. It’s a lot of information, and if you’re trying to make it more simple for people to wanna go to” [ID 07, office manager at an RCC^a^] More personalization and person-centered: “... but you can look at doing patient-centered language. As opposed to domestic violence or MAT, you could frame it as the—‘I’m not safe in my living environment’ or ‘I’m trying to get clean.’” [ID 12, physician at a hospital-based clinic]. Need for more specialized content: “... talking about concentration of medication and breastfeeding, I think that was a topic. I can’t remember if I read about that when I scrolled through. Moms ask, ‘Is this safe?’” [ID 16, instructor at a hospital-based clinic].	More educational content on how to manage triggers and cravings: “Maybe keeping track of how many triggers we have a day. If we have a bunch of cravings then we might need to change our surrounding” [ID32]. Easier navigation of app and ability to personalize content: “... curated information. directly linked resources curated to the individual. [For example], I click on the link fill out the assessment or forms and the app notifies me when the agency responds or at least reminds me to check my email for that response” [ID18]. More content and resources specific to pregnancy and parenting: “Medications that could help with stimulant usage during pregnancy” [ID44].
Favorite uMAT-R feature	Personalized features: “I definitely like the homepage. This is the how many days sober, and how many days of treatment? It walks you through. It gives you ... something extraneous of yourself that you can look at, ‘Okay, how am I doing? How long have I been in this process?’” [ID 11, behavioral health technician at an RCC]. Information about treatment and medication: “I did like the videos. I liked putting a face to the story. I did like the information about MAT ... it’s nice that they can get the information that MAT does work and it is a positive and not a negative” [ID01, certified peer support specialist at a hospital-based clinic for people in treatment for opioid use disorder during the postpartum period].	In-app messaging to e-coach: “Texting with counselor” [ID41] and “Messaging” [ID43]. Interactive features (eg, sober day tracker, in-app assessments): “Also, they had the days sober. I also like they had that on there too because I’ve been doing the track of how many days I’ve been sober” [ID09]. Educational and motivational information: “Motivational quotes and the simplicity of the lessons” [ID13].

^a^RCC: recovery community centers.

**Table 5:** Themes: e-coaching conversations and motivations for app use.

Categories	Theme and quote	
	Provider	Client
e-Coach conversations	Support to manage emotions and behavior: “When the baby’s screaming, you’re overwhelmed, you’re finding you’re getting evicted from your apartment, you’ve just lost your job, recently, you don’t have enough—when all of these stressors are compounding on you, what are you gonna do in that moment? [ID16, physician at a hospital-based clinic for people in treatment for opioid use disorder during the postpartum period]. Meeting basic needs: “Cause I think recovery is a really multifaceted goal, and for a lot of people the recovery part of that is ... just learning how to take care of themselves, and where to find good, affordable food ...” [ID06, coordinator at an RCC^a^]. Taking care of baby: “Well, lots of things. I can think of 100 things right now. If they have any extra resources. Does this spot on my baby look worrisome? Is it too cold to take him outside with a light blanket” [ID10, community outreach specialist at an RCC]. Weekly check-ins with e-coach: “It would probably be each week. A lot of my clients say they’re busy. They got things going on in their lives, so each week would be, preferably” [ID15, community health worker at agency for postpartum people and babies]. Frequency of messages depends on clientb: “I mean, everybody’s journey is different. One person may not utilize the messaging which the next person may utilize that strongly ...” [ID04, recovery coach at an RCC].	Setting and monitoring progress toward recovery goals: “about my progress, goals, or life concerns ...” [ID35]. Support with daily concerns (eg, substance use, mental health, and services): “About how to go certain ways with getting help that I need with my ADHD that I don’t get prescribed medicine for due to my past drug usage” [ID44]. At least weekly messages with e-coach: “maybe every 3 days, to serve as a reminder someone is always there to help or talk” [ID29].
Motivation to use uMAT-R	Receive emotional support: “[Speaking of the e-coach), ‘Oh, this is someone that hasn’t seen me, met me. They haven’t judged me on any preliminary things. They’re just someone here to listen and get to know me.’ That might even be a motivating factor” [ID11, behavioral health technician at an RCC]. Access to tools to reduce recovery barriers: “More resources and more tools you have to help [the better], especially with everybody on their phones, that’s why I kinda liked how [this app] is” [ID07, office manager at an RCC].	Access to resources about recovery and parenting: “This app helps me with all of these things cause I get to do surveys and answer questions about these things honestly, I love that I have endless resources to learn and read about that are specifically about my recovery” [ID37]. Flexible support and accountability from e-coach: “Coaching has helped” [ID41] and “Staying motivated and knowing that there’s always someone who is willing to help” [ID13]. Access to personalized features and educational content: “It motivates and educates me on this subject to stay clean & I already know about MAT and I study and discuss triggers in my daily groups” [ID13]. Personal goal of recovery and sobriety: “This pregnancy is my motivation I have to stay sober I do not want to lose my baby” [ID14]. Compensation for participating in study activitiesb: “Well right now it is the gift cards that’s motivating me because I don’t have a job currently so the money is helping me with baby food / supplies” [ID44].

^a^RCC: recovery community centers.

^b^Denotes minor theme.

#### Favorite uMAT-R Feature

Clients liked the in-app messaging feature, which allowed them to connect with the e-coach. Clients also enjoyed the interactive features of the app, such as the sober day tracker and the in-app assessments of current SUD symptoms and confidence in meeting basic needs. Clients liked the variety of information offered in the app, including the educational courses and daily motivational quotes that would appear on the app homepage.

Providers especially liked features that already seemed interactive and personalized for clients. These included the sober day tracker, the in-app messenger that allowed specialized support from the e-coach, and daily assessments about well-being and substance use. Providers conveyed satisfaction with the information about treatment and medication available in the app.

#### e-Coach Conversations

Clients shared that they had used the in-app messaging feature to receive help from the e-coach with setting and monitoring progress toward substance use recovery goals. Clients also contacted the e-coaches for support as daily concerns emerged, often related to their substance use and mental health and on how to engage with local recovery resources. Clients wanted to be able to converse with their e-coach at least once weekly, as many clients reported that they would prefer multiple check-ins per week.

Providers recommended certain topics of conversation that could occur between a client and an e-coach. Major themes included how e-coaches could help clients manage emotions and behavior, meet basic needs through accessing resources, and provide education to help care for their baby. Providers noted the key role of the e-coach in helping clients access resources to support basic needs, take care of their babies, and manage everyday stressors. Weekly check-ins were endorsed by providers as the ideal frequency for e-coaches to check in on their clients; the frequency of messages being dependent on the client and their stage in recovery was identified as a minor theme.

#### Motivation to Use uMAT-R

Clients were motivated to use the app, as it provided them with access to local resources for recovery, parenting, and managing a family. Clients appreciated that the e-coaching feature allowed them to benefit from flexible support that fits their schedule and that the e-coach provided motivation to continue working toward recovery. Personal goals of achieving recovery for themselves and their loved ones also served as motivation to use the app. Finally, receiving compensation for participation in the app and the related study was a minor theme among clients, as gift cards could be used to purchase items for their baby and family.

Two major themes that providers noted as motivators for clients to use the uMAT-R app included emotional support available through the e-coach and access to tools to reduce recovery barriers. An in-app directory of recovery resources and services, educational content, and information were cited by providers as motivating factors for app use. Most providers felt that their clients would use the app and felt that the app offered important features that could help facilitate recovery.

#### What to Change Within the App

Clients voiced a need for an app that was easy to navigate, such as being able to “skip” through content, and to have options for personalization of content (eg, access to more assessments once completed). Clients also mentioned wanting extensive educational content in the app about how to manage triggers and cravings related to substance use and content and resources that were specific to being pregnant and being a parent—both in the context of SUD recovery and in general. For example, clients wanted more information on how to handle potential issues with child custody and local resources for childcare. A minor theme included being able to use the messaging feature to connect with other people who were working toward recovery, not just the e-coaches.

Similarly, providers agreed that the app content could be more streamlined (eg, having courses better organized to support ease of use among clients) and be tailored to use more person-centered language. Suggestions such as building a separate directory for this population, being mindful of the use of clinical and potentially stigmatizing jargon, involving the e-coach in the curation of content, and including topics such as breastfeeding while taking MOUD were all provided as ways to improve the app. Pairing of the goals feature with e-coaching support was a minor theme identified to better support clients.

### Post Hoc Analysis

We analyzed the demographic characteristics of clients who completed surveys but did not log into the app (Multimedia Appendix 3).

## Discussion

### Principal Findings

This study contributes to needed research on formative evaluations that include both pregnant and postpartum people and substance use providers’ perspectives on mHealth as part of substance use recovery while also addressing gaps in key informant representation in addiction science research. This study sought to include underserved and high-need client populations in user-centered design for digital interventions [35,36] (eg, unemployed and less formal education) [37] and less often represented health care providers, such as peers, community health workers, and behavioral health technicians. Additionally, this study sought to identify similarities and differences in feedback on the proposed mHealth tool among these high-need clients and their providers to better address the diverse needs of the pregnant and postpartum recovery community. The perspectives provided by both groups aligned on themes related to the usability and compatibility of the uMAT-R app for SUD recovery, particularly emphasizing the utility of the app’s messaging feature, which facilitated supportive and knowledgeable conversations with an e-coach. They also mirrored each other’s praise for the inclusion of interactive features, such as a sober day tracker and an extensive psychoeducational library, which allowed for personalization. Themes from the pregnant and postpartum clients provided a more nuanced understanding of the app’s compatibility with their specific needs, and they provided suggestions for more content on parenting and new capabilities to message with peers within the app. Meanwhile, the health care providers offered multifaceted insights on the mHealth intervention, not only informed by their professional practices with diverse client bases but often also their own personal backgrounds as people with lived experience in recovery.

The e-coaching modality was one of the most liked features of the mobile app. This finding contrasts with common perceptions that communication only via text may jeopardize trust and rapport and reduce app engagement [38,39]. Additionally, both provider and client findings suggest that mHealth interventions, such as uMAT-R, may be especially useful in providing readily available daily support and recovery-related problem-solving to clients and help to facilitate linkage to community-based resources. These findings also align with transportation being reported by both groups as a major barrier to health care appointments. This is in line with general sentiments within the broader addiction research community that digital interventions can improve overall access to care for SUDs [40].

With respect to the usability of the mobile app, both clients and providers requested that content be easier to search through and find specific information of interest. Ease of navigation is a central aspect of app usability. Similar to prior qualitative studies [41,42], findings from both the providers and clients also highlighted the importance of intervention personalization, as we continue to iterate versions of the app. For example, in the future, we may be able to add more tailored features such as providing automated or coach-curated educational content based on the client’s assessment responses or on conversation topics with the e-coach.

In terms of compatibility with the mobile app, both clients and providers shared that regular support with daily recovery stressors and parenting and access to resources are key motivators to using the app. Clients reported that gift card compensation for participation was an additional motivator. One client wanted more educational content that was not tied directly to being in recovery from SUD. This comment aligns with previous research that found that many individuals viewed their recovery as an opportunity to also return to “normalcy” [43]. End users may appreciate a mobile app that focuses on their other life roles (eg, parent, spouse, and employee) and builds leisure skills. Indirectly, this comment may also align with a theme from the providers that language within the app be updated to be more person-centered versus clinical.

Negative cases identified during analyses (as mentioned in the Methods section) provided insight into how mobile app preferences can be very personal. For some, writing by hand may be a more multisensory and emotional experience preferred to typing with a digital tool. Additionally, an mHealth app may be more useful for people at the beginning of their SUD recovery when they may have fewer supports and resources than for individuals who are at later stages in their recovery. Available literature suggests that people earlier in recovery benefit from more intensive and structured support compared to those later in recovery who may have already developed long-standing support systems [44]. Future iterations of this study may explore how to personalize mobile app content and programming to better suit individual factors, such as stage in recovery.

Study findings may have valuable implications for sustaining engagement among high-need and at-risk pregnant and postpartum clients in digital interventions and SUD treatment. The active client subgroup reported various barriers to treatment continuation and recovery, such as missing at least 1 appointment in the past month and having more than 1 phone in the past year (a proxy for interruption in communication with providers and, thus, care). At the same time, this subgroup included individuals with overdose risk factors (eg, in treatment for opioid and/or stimulant and no MOUD prescription use among the majority in treatment for opioids), further emphasizing the potential value of the participants’ perspectives. Additionally, the inclusion of providers with lived experience of SUD and recovery, and who work at different stages in the continuum of care, may have provided diverse perspectives, which studies that only include one provider discipline may miss [45].

Post hoc, the authorship team decided to analyze the survey responses of the inactive clients to better understand potential gaps in app use (Multimedia Appendix 3). Our subsample of active clients was predominantly White, while, among those who were inactive in the app (n=8), there was a higher proportion of Black individuals. Additionally, fewer inactive clients had formal education beyond a high school diploma or a General Educational Development test, and more had families of multiple children. These findings suggest that the racial and educational disparities often observed in in-person care for SUD may also translate to digital interventions and tools [40]. Thus, mHealth and substance use treatment scholars need to identify strategies that can ameliorate socioecological barriers. Mobile app content may need to be updated to be more inclusive and appropriate for different levels of learning (eg, reading level) and households (eg, shorter lessons and assessments for busy parents and use of videos versus only PDFs and PPTs for providing psychoeducation). Some individuals may need additional support with addressing gaps in digital literacy skills (eg, using the mobile app features) and digital access (eg, purchasing cellular data and locating public Wi-Fi). Across participant groups, transportation stood out as a crucial barrier to receiving SUD treatment.

Including providers’ perspectives when developing and refining mHealth tools such as uMAT-R can validate the ideas and reported needs of pregnant and postpartum participants. A strength of our study is that nearly half of the providers shared that they were in recovery from SUD themselves; these individuals could speak to lived experience as both a client and a provider. Synergy between mHealth development teams and providers is also important for establishing a united front when using mHealth in the real world. Trusted providers can often serve as models or gatekeepers for what resources and tools “work” or “do not work” in the eyes of clients. Providers can offer insight on mHealth tools informed by their experience serving adults with SUD and bridge the gap between active, long-term mHealth use and passive, short-term app engagement.

### Limitations

Our study’s findings should be interpreted within the limitations of its design. The transferability of the findings may be limited by the sociodemographic profile of the clients and providers. The subsamples of clients and providers predominantly identified as cisgender and White. Reflecting the nature of the pilot study, the sample size was small and was solicited across one region of the Midwest. Pregnant clients were also only in the second and third trimesters, as these specific clients and those who were postpartum were more likely to seek care at the participating recruitment sites.

Additionally, we acknowledge changes to the study research team through the course of the study and the need to pivot from a “live” interview to an open-ended survey to best suit participants’ schedules and needs. Changes to study methods always have the potential to impact the dependability and the confirmability of the research findings. To mitigate these risks, all interviewers provided an audit via an interview summary after each interview, and the coders clearly documented their coding process in Excel.

### Conclusions

This study included qualitative responses from pregnant and postpartum people and SUD providers. Both groups of key informants were asked questions about the usability and compatibility of an mHealth intervention to support SUD recovery. While both groups described similar themes, pregnant and postpartum participants described specific nuances in their preferences for the mHealth intervention that were not shared by the providers. e-Coaching and interactive features were the most well-received components, and navigability and personalization of the mHealth intervention were highlighted as key aspects to explore in future work to increase the uptake and impact of such digital tools. Additionally, baseline data from participants who did not use the app offered insight into potential barriers to use (eg, structural racism in health care, less formal education, and taking care of family and other children), reinforcing that pregnancy and the postpartum period is a high-need time, in which innovative approaches to outreach, engagement, and retention in care (both digital and in person) are needed. Overall, both participants’ and providers’ feedback suggest the feasibility of using mHealth to support SUD recovery among pregnant and postpartum parents, and the usability and compatibility factors identified in this study should be considered when designing and refining mHealth interventions to meet the unique needs of this population.

## Appendix

Multimedia Appendix 1 uMAT-R app, including home page, course library, and messaging feature, respectively.

Multimedia Appendix 2 Pregnant and postpartum inactive participant demographics (n=8).

Multimedia Appendix 3 Evaluation of study quality and rigor.

## Abbreviations

IRB: institutional review board
mHealth: mobile health
MOUD: medication for opioid use disorder
REDCap: Research Electronic Data Capture
SUD: substance use disorder

## Data Availability

The datasets generated or analyzed during this study are not publicly available due to the sensitive nature of the data and to protect participant confidentiality and privacy. Data are available from the corresponding author on reasonable request.

## References

[1] Bhadra-Heintz Nia M, Garcia Stephanie, Entrup Parker, Trimble Candice, Teater Julie, Rood Kara, Trent Hall O (2023). Years of life lost due to unintentional drug overdose among perinatal individuals in the United States. Sex Reprod Healthc.

[2] Schiff DM, Nielsen T, Terplan M, Hood M, Bernson D, Diop H, Bharel M, Wilens TE, LaRochelle M, Walley AY, Land T (2018). Fatal and nonfatal overdose among pregnant and postpartum women in Massachusetts. Obstet Gynecol.

[3] Smid MC, Stone NM, Baksh L, Debbink MP, Einerson BD, Varner MW, Gordon AJ, Clark EAS (2019). Pregnancy-associated death in Utah: contribution of drug-induced deaths. Obstet Gynecol.

[4] Fuchs JR, Schiff MA, Coronado E (2023). Substance use disorder-related deaths and maternal mortality in New Mexico, 2015-2019. Matern Child Health J.

[5] Rodriguez CE, Klie KA (2019). Pharmacological treatment of opioid use disorder in pregnancy. Semin Perinatol.

[6] Minozzi S, Amato L, Jahanfar S, Bellisario C, Ferri M, Davoli M (2020). Maintenance agonist treatments for opiate-dependent pregnant women. Cochrane Database Syst Rev.

[7] Choi S, Rosenbloom D, Stein MD, Raifman J, Clark JA (2022). Differential gateways, facilitators, and barriers to substance use disorder treatment for pregnant women and mothers: a scoping systematic review. J Addict Med.

[8] Volkow ND, Blanco C (2023). Substance use disorders: a comprehensive update of classification, epidemiology, neurobiology, clinical aspects, treatment and prevention. World Psychiatry.

[9] Tsuda-McCaie F, Kotera Y (2022). A qualitative meta-synthesis of pregnant women's experiences of accessing and receiving treatment for opioid use disorder. Drug Alcohol Rev.

[10] Cerdá M, Krawczyk N (2020). Pregnancy and access to treatment for opioid use disorder. JAMA Netw Open.

[11] Ryu S (2012). Book Review: mHealth: new horizons for health through mobile technologies: based on the findings of the second global survey on eHealth (Global Observatory for eHealth Series, Volume 3). Healthc Inform Res.

[12] Gustafson DH, Landucci G, McTavish F, Kornfield R, Johnson RA, Mares ML, Westergaard RP, Quanbeck A, Alagoz E, Pe-Romashko K, Thomas C, Shah D (2016). The effect of bundling medication-assisted treatment for opioid addiction with mHealth: study protocol for a randomized clinical trial. Trials.

[13] Feroz A, Perveen S, Aftab W (2017). Role of mHealth applications for improving antenatal and postnatal care in low and middle income countries: a systematic review. BMC Health Serv Res.

[14] Dopp AR, Parisi KE, Munson SA, Lyon AR (2019). A glossary of user-centered design strategies for implementation experts. Transl Behav Med.

[15] Shorey S, Ng ED (2019). Evaluation of mothers' perceptions of a technology-based supportive educational parenting program (Part 2): qualitative study. J Med Internet Res.

[16] Sugarman DE, Meyer LE, Reilly ME, Rauch SL, Greenfield SF (2021). Exploring technology-based enhancements to inpatient and residential treatment for young adult women with co-occurring substance use. J Dual Diagn.

[17] Raynor P, Corbett C, Prinz R, West D, Litwin A (2022). Using community-based participatory methods to design a digital intervention for mothers with substance use disorders: qualitative results from focus group discussions. Perspect Psychiatr Care.

[18] (1998). ISO/TC 159/SC 4: ergonomics of human-system interaction (subcommittee). Ergonomic requirements for office work with visual display terminals (VDTs): guidance on usability. International Organization for Standardization.

[19] (2008). ISO/IEC 25012:2008(en): Software engineering—Software product Quality Requirements and Evaluation (SQuaRE)—Data quality model. International Organization for Standardization.

[20] Or CK, Holden RJ, Valdez RS, Duffy VG, Ziefle M, Rau PLP (2023). Human factors engineering and user-centered design for mobile health technology: enhancing effectiveness, efficiency, and satisfaction. Human-Automation Interaction: Mobile Computing.

[21] Eswaran V, Dong F, Li X, Szlyk HS, Dell NA, Kasson E, Williams J, Cavazos-Rehg PA (2026). Leveraging a digital health intervention to improve recovery outcomes among people with substance misuse experiencing housing insecurity. Drug Alcohol Depend.

[22] Szlyk HS, Dell NA, Li X, Winograd RP, Cavazos-Rehg P (2025). Psychosocial outcomes associated with mobile health app use among Medicaid recipients who use substances. J Soc Work Pract Addict.

[23] Cloud W, Granfield R (2008). Conceptualizing recovery capital: expansion of a theoretical construct. Subst Use Misuse.

[24] Rollnick S, Miller WR (2009). What is motivational interviewing?. Behav Cogn Psychother.

[25] Moumane K, Idri A, Abran A (2016). Usability evaluation of mobile applications using ISO 9241 and ISO 25062 standards. Springerplus.

[26] Creswell JW, Miller DL (2000). Determining validity in qualitative inquiry. Theory Pract.

[27] Downe-Wamboldt B (1992). Content analysis: method, applications, and issues. Health Care Women Int.

[28] Graneheim UH, Lundman B (2004). Qualitative content analysis in nursing research: concepts, procedures and measures to achieve trustworthiness. Nurse Educ Today.

[29] Zhang Y, Wildemuth BM (2005). Qualitative analysis of content by. Human Brain Mapping.

[30] Lincoln YS, Guba EG (1985). Naturalistic Inquiry.

[31] Emigh RJ (1997). The power of negative thinking: the use of negative case methodology in the development of sociological theory. Theory Soc.

[32] Cachelin FM, Striegel-Moore RH (2006). Help seeking and barriers to treatment in a community sample of Mexican American and European American women with eating disorders. Int J Eat Disord.

[33] Key Substance Use and Mental Health Indicators in the United States: Results from the 2023 National Survey on Drug Use and Health.

[34] Fentem A, Riordan R, Doroshenko C, Li X, Kasson E, Banks D, Winograd RP, Cavazos-Rehg P (2023). Impact of the COVID-19 pandemic on burnout and perceived workplace quality among addiction treatment providers. Addict Sci Clin Pract.

[35] Krukowski RA, Ross KM, Western MJ, Cooper R, Busse H, Forbes C, Kuntsche E, Allmeta A, Silva AM, John-Akinola YO, König LM (2024). Digital health interventions for all? Examining inclusivity across all stages of the digital health intervention research process. Trials.

[36] Bazzano AN, Noel LA, Patel T, Dominique CC, Haywood C, Moore S, Mantsios A, Davis PA (2024). Correction: Improving the engagement of underrepresented people in health research through equity-centered design thinking: qualitative study and process evaluation for the development of the grounding health research in design toolkit. JMIR Form Res.

[37] Straand IJ, Baxter KA, Følstad A (2024). Remote inclusion of vulnerable users in mhealth intervention design: retrospective case analysis. JMIR Mhealth Uhealth.

[38] Aronowitz SV, Engel-Rebitzer E, Dolan A, Oyekanmi K, Mandell D, Meisel Z, South E, Lowenstein M (2021). Telehealth for opioid use disorder treatment in low-barrier clinic settings: an exploration of clinician and staff perspectives. Harm Reduct J.

[39] Glass JE, Matson TE, Lim C, Hartzler AL, Kimbel K, Lee AK, Beatty T, Parrish R, Caldeiro RM, Garza McWethy A, Curran GM, Bradley KA (2021). Approaches for implementing app-based digital treatments for drug use disorders into primary care: a qualitative, user-centered design study of patient perspectives. J Med Internet Res.

[40] Hampton J, Mugambi P, Caggiano E, Eugene R, Valente A, Taylor M, Carreiro S (2024). Closing the digital divide in interventions for substance use disorder. J Psychiatr Brain Sci.

[41] Peng W, Kanthawala S, Yuan S, Hussain SA (2016). A qualitative study of user perceptions of mobile health apps. BMC Public Health.

[42] Alqahtani F, Winn A, Orji R (2021). Co-designing a mobile app to improve mental health and well-being: focus group study. JMIR Form Res.

[43] Schoenberger SF, Park TW, dellaBitta V, Hadland SE, Bagley SM (2022). "My Life Isn't Defined by Substance Use": recovery perspectives among young adults with substance use disorder. J Gen Intern Med.

[44] Webb L, Clayson A, Duda-Mikulin E, Cox N (2022). ' I' m getting the balls to say no': trajectories in long-term recovery from problem substance use. J Health Psychol.

[45] McKenna SA, Iwasaki PG, Stewart T, Main DS (2011). Key informants and community members in community-based participatory research: one is not like the other. Prog Community Health Partnersh.

