# Supplementary figures and images for: Comparing Pregnant and Postpartum Client and Provider Feedback on a Digital Health Intervention for Substance Use Recovery: User-Centered Design Approach
Source: JMIR Form Res. 2026 Mar 9;10:e86255. doi: 10.2196/86255 (PMC13010078; doi:10.2196/86255)

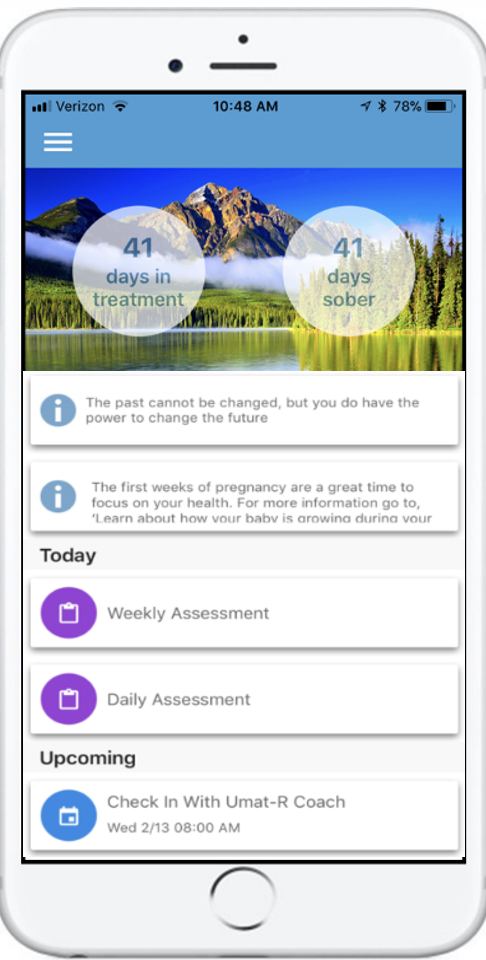

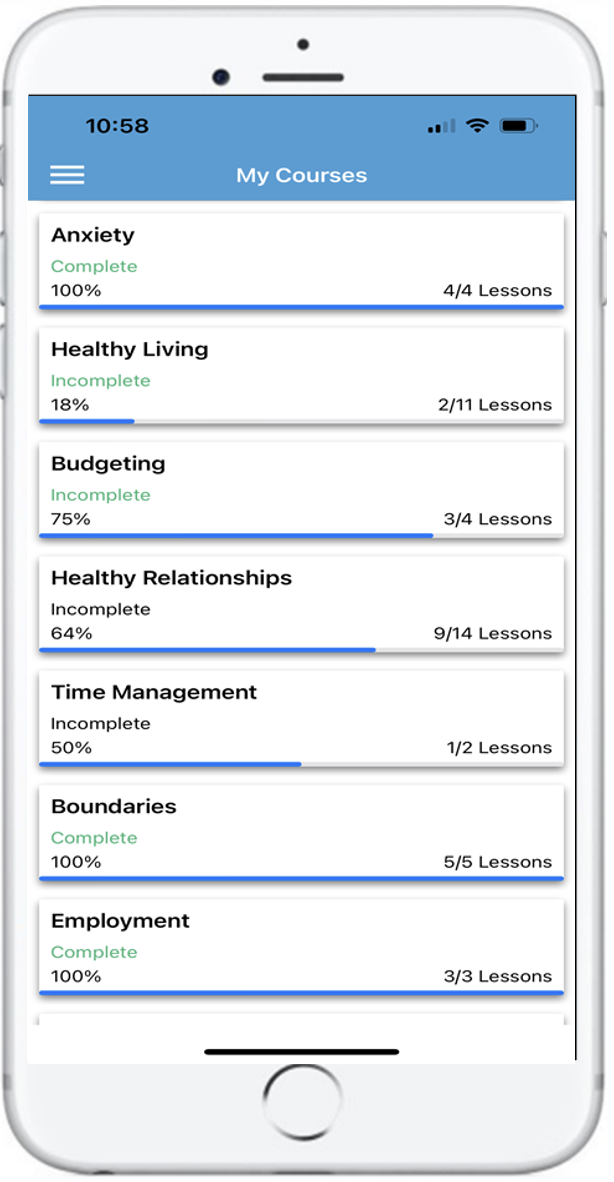

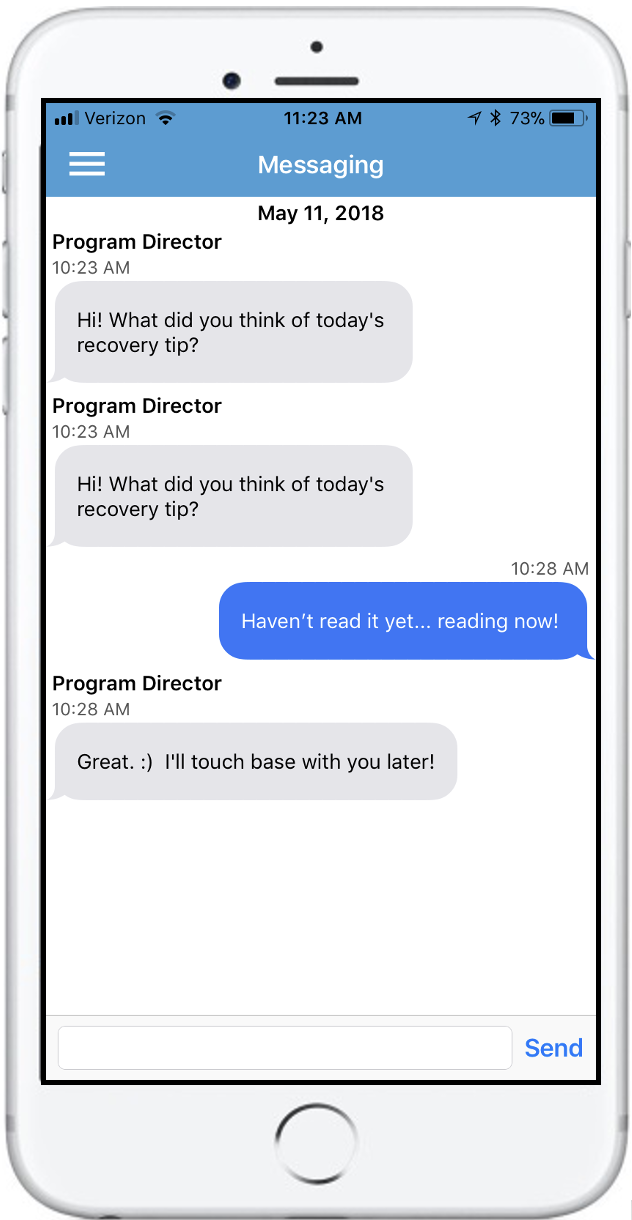

Supplement: Multimedia Appendix 1 [file formative_v10i1e86255_app1.docx]
