# Supplementary material for: Comparing Pregnant and Postpartum Client and Provider Feedback on a Digital Health Intervention for Substance Use Recovery: User-Centered Design Approach
Source: JMIR Form Res. 2026 Mar 9;10:e86255. doi: 10.2196/86255 (PMC13010078; doi:10.2196/86255)
Supplement: Multimedia Appendix 2 [file formative_v10i1e86255_app2.docx]

|  | n (%) |
| --- | --- |
| **Substance use** |  |
| Opioid Use | 2 (25) |
| Stimulant Use | 2 (25) |
| Opioid and Stimulant Use | 4 (50) |
| **Gender identity** |  |
| Women | 8 (100) |
| **Race/Ethnicity** |  |
| White | 3 (37.5) |
| Black/African American | 5 (62.5) |
| **Education level** |  |
| 12^th^ Grade/No Diploma | 2 (25) |
| GED/High School Diploma | 4 (50) |
| Some college or higher | 2 (25) |
| **Employment Status** |  |
| Employed Full-Time | 2 (25) |
| Employed Part-Time | 0 |
| Unemployed | 6 (75) |
| **Pregnant/Postpartum** |  |
| Pregnant | 4 (50) |
| Postpartum | 4 (50) |
| **MOUD** |  |
| Prescribed MOUD? | 0 |
| **Health Insurance** |  |
| Medicaid | 6 (75) |
| Private Health Insurance | 1 (12.5) |
| No Insurance | 1 (12.5) |
| **Number of Children** |  |
| 1 | 2 (25) |
| 2 | 2 (25) |
| 3 | 4 (50) |
| 4+ | 0 |
| **Number of Phones in Past 12 Months** |  |
| 1 | 3 (37.5) |
| 2 | 3 (37.5) |
| 3+ | 2 (25) |
| **Number of Phone Numbers in Past 12 Months** |  |
| 1 | 3 (37.5) |
| 2 | 4 (50) |
| 3+ | 1 (12.5) |
| **Treatment Status** |  |
| Currently in Treatment | 6 (75) |
| Not Currently in Treatment | 2 (25) |
| **Any Missed Appointments in Past Month** |  |
| Yes | 4 (50) |
| No | 4 (50) |
