# Supplementary material for: Comparing Pregnant and Postpartum Client and Provider Feedback on a Digital Health Intervention for Substance Use Recovery: User-Centered Design Approach
Source: JMIR Form Res. 2026 Mar 9;10:e86255. doi: 10.2196/86255 (PMC13010078; doi:10.2196/86255)
Supplement: Multimedia Appendix 3 [file formative_v10i1e86255_app3.docx]

| Criteria | Strategy | Study Examples |
| --- | --- | --- |
| Credibility | Prolonged Engagement | The interviewer of the providers had met some of the provider participants previously, as they are all part of a small, urban recovery service network. |
|  | Negative case analysis | The analysts identified negative cases which allowed them to better understand the needs of future clients and mHealth end users. |
|  | Triangulation | The study compared perspectives of study participants who had different viewpoints and experiences. |
| Transferability | Thick Descriptions | Authors put the study into context by providing detailed descriptions of the parent study, the study setting, and the data collection and analysis process. Provider and client responses are reviewed separately and in comparison. |
|  | Sampling Strategies | The authors used purposive and snowball sampling, which also mirrors how potential clients of the parent study are engaged in behavioral services in the region. Provider participants were recruited from a large network of providers and scholars of SUD treatment, who represent various disciplines and service roles that are representative of the US-based behavioral healthcare system. |
| Dependability | Methodological Documentation | The procedures of the study, including approaches used to engage both providers and clients, are documented in the methods section of the manuscript. |
|  | Inquiry Audit | Some members of the authorship team were not a part of the research team and could provide additional evaluation of the research methods and findings. |
| Confirmability | Audit Trails | The interviewer for the provider participants completed an interview summary after each interview, which recorded overarching themes of the interview, how the interview compared to others, and points to consider for future interviews |
|  | Analyst Triangulation | Coders of the qualitative data met regularly to identify different perspectives on the data and to work towards a consensus on coding of the datasets. |
